# Application of Elongation Method-Based Alternating Property Optimization: (Hyper)polarizability of Substituted Polyfuran

**DOI:** 10.3390/molecules30112409

**Published:** 2025-05-30

**Authors:** Shichen Lin, Yuuichi Orimoto, Yuriko Aoki

**Affiliations:** 1 Interdisciplinary Graduate School of Engineering Sciences, Kyushu University, 6-1 Kasuga-Park, Fukuoka 816-8580, Japan; lin.shichen.312@s.kyushu-u.ac.jp; 2 Department of Molecular and Material Sciences, Interdisciplinary Graduate School of Engineering Sciences, Kyushu University, 6-1 Kasuga-Park, Fukuoka 816-8580, Japan; orimoto.yuuichi.888@m.kyushu-u.ac.jp

**Keywords:** the elongation method, non-linear optics, polyfurans

## Abstract

The alternating property optimization (POPT) approach was employed to optimize the (hyper)polarizabilities of donor–acceptor-substituted polyfuran (PFu). The capability of the alternating POPT to design systems with specific properties was further demonstrated by the results, and its accuracy was validated. In both the $\alpha_{zz}$-maximizing and $\alpha_{zz}$-minimizing POPT, the selected monomers exhibited clear and consistent patterns, which may provide useful insights for the future design of PFu-based materials. Combined with the POPT results, the comparison of CPU time between the alternating POPT and the existing simultaneous POPT further demonstrated the reliability and efficiency of alternating POPT while handling systems growing along multiple directions.

## 1. Introduction

The elongation (ELG) method is a linear-scaling ab initio approach for treating large systems [1,2]. It theoretically simulates a polymerization process, where monomers are added one by one to the terminal of a small starting cluster and calculated at each ELG step, ultimately yielding the electronic structure of the target polymer. In each ELG step, due to the utilization of the regional localized molecular orbital (RLMO) [3], the Self-Consistent Field (SCF) calculation is required only for the subsystem containing the newly added monomer at the terminal, referred to as the “interactive space”. The remaining units can be treated as the frozen region, whose electronic structures are obtained from previous ELG steps. Therefore, the computational data from each ELG step can be stored on disk. When a different attacking monomer is added in the next ELG step, the corresponding calculation can be carried out by retrieving the stored data [4]. This feature of the ELG method is referred to as “restart”. The “restart” feature enables the development of the ELG-based property optimization (POPT) method [5]. This process facilitates the automated design of DNA materials with specific Non-Linear Optical (NLO) properties from one terminal to the other. At each POPT cycle, a base pair is selected and retained based on an evaluation function related to the system’s (hyper)polarizabilities, which are calculated using the ELG-Finite Field (ELG-FF) method [6,7,8]. ELG-based POPT has been reported to be significantly more efficient than the POPT approach based on conventional SCF calculations applied to the entire system [5]. To design systems with multiple growth directions, an alternating ELG-based POPT approach has recently been introduced, allowing the POPT process to proceed alternately at different terminals. Compared to the simultaneous POPT using existing center-to-terminal (C2T) ELG, in which monomers are selected simultaneously in all terminals, the alternating POPT further improves efficiency by significantly reducing the number of combinations of monomers and basis functions involved in the calculations [9]. Given these methodological advances, the alternating POPT scheme becomes a promising tool for designing complex functional materials, including conjugated polymers.

Conjugated polymers are carbon-based macromolecules in which the valence π-electrons are delocalized along the molecular backbone. Their unique linear optical and NLO properties have attracted considerable attention from researchers. They are widely regarded as promising materials in the electronics industry, with potential applications in light-emitting devices, photovoltaic devices, and NLO devices [10,11]. Many insightful studies, both theoretical and experimental, have been conducted on these properties of conjugated polymers [12,13,14,15,16,17,18,19,20,21,22,23,24,25,26,27,28,29,30,31,32]. Through experiments, Marder et al. demonstrated that controlling bond length alternation and aromaticity in push–pull polyenes can significantly enhance the first hyperpolarizability [12]. Cheng et al. systematically studied a wide range of donor–acceptor π-conjugated systems, revealing how various factors such as side-group substitutions and aromaticity affect the NLO properties [13,14]. Several studies, including those by Samuel et al., have discussed the chain-length dependence and saturation behavior of NLO properties in conjugated polymers from both theoretical and experimental perspectives [16,17,18]. Brédas and co-workers provided a comprehensive quantum-chemical framework for understanding the excited-state electronic structure of conjugated oligomers and polymers, highlighting its central role in optical absorption, emission, and NLO phenomena [20]. Tretiak et al. introduced a conformational dynamics method for simulating excited-state molecular dynamics in π-conjugated systems, enabling the analysis of time-dependent photoexcitation and optical spectroscopy [21]. Given the efficiency and accuracy of the ELG-based POPT method, it is feasible to design large conjugated polymers with specific NLO properties. In previous work, the alternating POPT approach has already been applied to the design of polydiacetylene [9]. Polyfuran (PFu), as one of the electronically conducting organic polymers [33,34], has attracted considerable interest due to its NLO properties [35,36], thereby making it a suitable candidate for the application of alternating POPT. This work aims to further demonstrate the capability of alternating POPT by designing donor–acceptor-substituted PFus and to provide new design strategies for future PFu-based materials.

## 2. Results

### 2.1. Result of Alternating POPT

Three alternating POPT processes aimed at maximizing the $\alpha_{zz}$, $|\beta_{zzz}|$, and $\gamma_{zzzz}$ of donor–acceptor-substituted PFus were conducted separately. In contrast, three POPT processes aimed at minimizing the $\alpha_{zz}$, $|\beta_{zzz}|$, and $\gamma_{zzzz}$ were also conducted. Since $\beta_{zzz}$ may deviate from zero in either a positive or negative direction, the absolute value $|\beta_{zzz}|$ is employed for simplicity and clarity in analysis. All six POPT processes started from an initial cluster containing 17 furan units. In the original version of the ELG program, the appropriate size of the interactive space is automatically determined from two units as the minimum size required to maintain accuracy [2]. However, during the current development stage of the alternating ELG-based POPT method, the size of the interactive space was fixed to 8 units in all ELG calculations. This value was determined through an iterative testing procedure introduced in our previous work [9], aiming to balance the accuracy and efficiency of ELG-FF calculations. This procedure is briefly described in Appendix B. In each POPT process, 8 D-furan and 8 A-furan monomers were incrementally added to both sides of the initial furan cluster in a total of 16 cycles.

**Figure 1:**
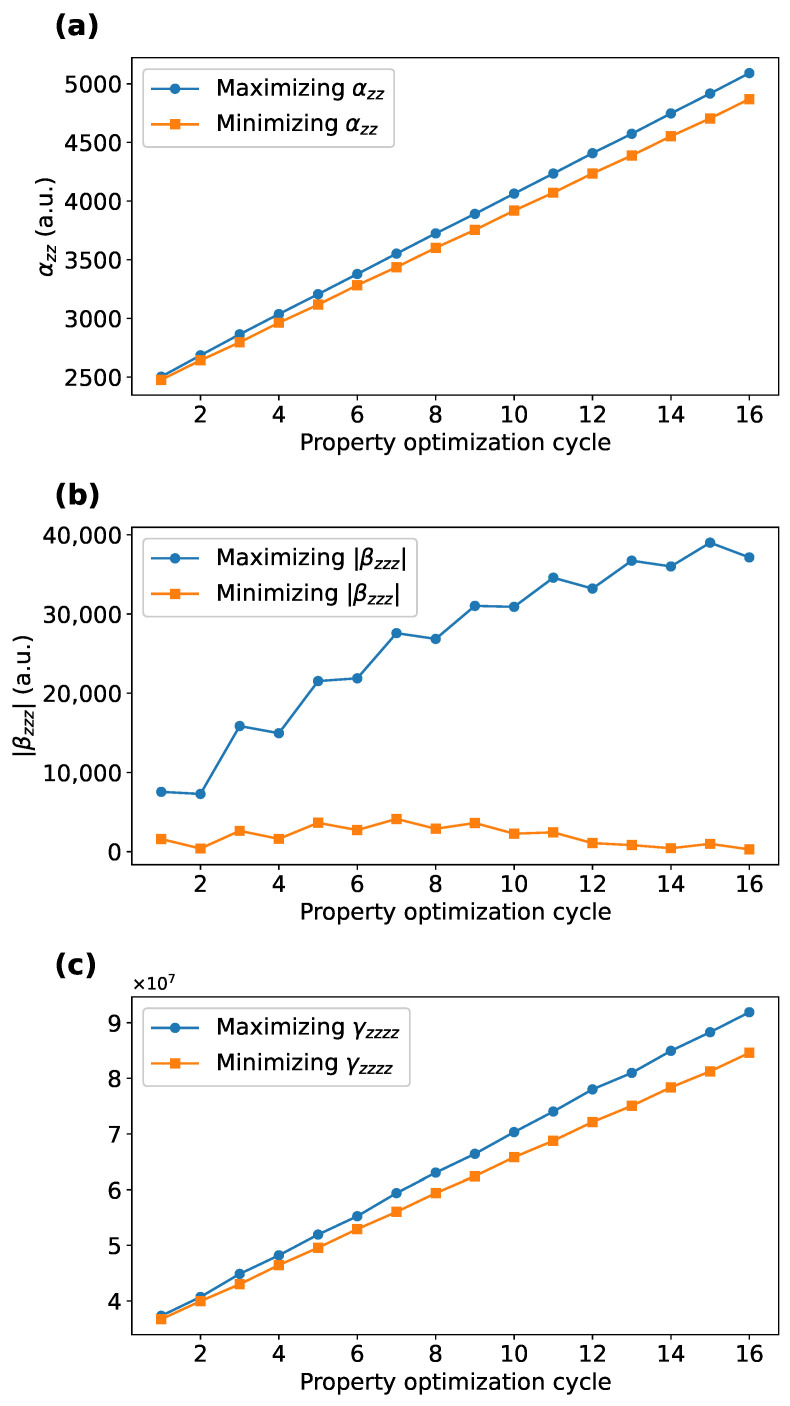
Comparison of the target properties of the resulting PFu at each POPT cycle, under conditions of maximizing and minimizing the respective properties: (**a**) $\alpha_{zz}$; (**b**) $|\beta_{zzz}|$; (**c**) $\gamma_{zzzz}$.

To assess the capability of alternating POPT, the POPT results aimed at maximizing and minimizing the particular (hyper)polarizability were compared. During the POPT process aimed at maximizing and minimizing $\alpha_{zz}$, the magnitude of $\alpha_{zz}$ for the resulting PFu after selecting a monomer in each cycle is compared in panel (a) of Figure 1. Similarly, the comparisons for $|\beta_{zzz}|$ and $\gamma_{zzzz}$ are presented in panels (b) and (c) of Figure 1, respectively.

Compared to the POPT process aimed at minimizing the PFus’ (hyper)polarizabilities, the POPT processes targeting the maximization of $\alpha_{zz}$, $|\beta_{zzz}|$, or $\gamma_{zzzz}$ demonstrate significantly faster rates of increase in their respective targeted physical quantities, especially for $|\beta_{zzz}|$. This underscores the capability of alternating POPT in designing systems with specific NLO properties, as also observed in our previous work [9].

Table 1 presents the D-/A-furan monomers selected in each cycle of the POPT processes. It is worth noting that the selected types of monomers exhibit a clear trend in the POPT concerning $\alpha_{zz}$. In the $\alpha_{zz}$-maximizing POPT, only furan monomers substituted with formyl and sulfhydryl groups are selected. In contrast, in the $\alpha_{zz}$-minimizing POPT, only furan monomers substituted with fluoro and hydroxyl groups are selected. In the POPT processes for $|\beta_{zzz}|$ and $\gamma_{zzzz}$, the selection patterns of monomers are less uniform, but some local trends can still be identified. In the $|\beta_{zzz}|$-maximizing POPT, amino-substituted furan monomers tend to be preferentially incorporated over other D-furan monomers. When the PFu chain grows longer (starting from the 9th POPT cycle), nitro-substituted furan monomers are more frequently selected compared to other A-furan monomers. In the $|\beta_{zzz}|$-minimizing POPT, fluoro-substituted furan monomers exhibit a higher likelihood of being incorporated than other A-furan monomers. In the $\gamma_{zzzz}$-minimizing POPT, fluoro-substituted furan monomers are also preferentially selected over other A-furan monomers. Moreover, starting from the sixth POPT cycle, hydroxyl-substituted furan monomers are favored over other D-furan monomers. This observation may provide new insights for the future development of PFu-based NLO materials.

### 2.2. Performance of Alternating POPT

To further validate the accuracy of the alternating POPT, (hyper)polarizabilities of PFus obtained after selecting the appropriate monomer in each POPT cycle of the $\alpha_{zz}$ maximization process were recalculated using the conventional FF method based on SCF calculations on the entire PFu. For each recalculated PFu, the errors per atom in (hyper)polarizabilities are evaluated using Equation (1) (with $\alpha_{zz}$ as an example) and illustrated in Table 2.

(1) $$Err\left(\alpha_{zz}\right)=\frac{|\alpha_{zz}\left(conv\right)-\alpha_{zz}\left(elg\right)|}{N_{atom}\times \alpha_{zz}\left(conv\right)}\times 100\%$$

where $\alpha_{zz}\left(conv\right)$ represents the value of $\alpha_{zz}$ obtained from conventional FF calculation, and $N_{atom}$ denotes the total number of atoms in the recalculated PFu. $\alpha_{zz}\left(elg\right)$ is the value of ELG-FF-calculated $\alpha_{zz}$ for the entire recalculated PFu.

As a numerical method, the FF approach introduces fluctuations in the computed errors of (hyper)polarizabilities, particularly for higher-order properties such as $\beta_{zzz}$ and $\gamma_{zzzz}$. Nevertheless, the (hyper)polarizability errors per atom obtained from the ELG-FF calculations remained at a relatively low level, with the maximum error staying around 0.01% per atom, which is on the same order of magnitude as reported in the previous study [9]. This once again confirms the accuracy of the alternating POPT approach.

**Figure 2:**
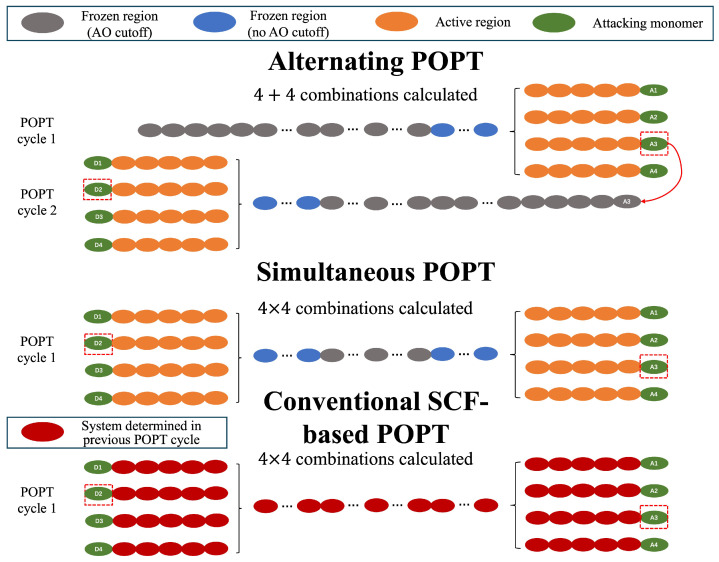
Comparison among alternating POPT, simultaneous POPT, and conventional SCF-based POPT in selecting a new monomer along the two elongation directions. The monomer(s) selected for providing the optimal property in a specific POPT cycle are highlighted with red dashed boxes.

In previous studies, the alternating POPT has been demonstrated to be more efficient than both the conventional SCF-based POPT [5], as well as the existing simultaneous POPT [9], in which monomers are simultaneously attached to both terminals of the system [37,38]. This efficiency advantage has been thoroughly discussed in previous work, both theoretically and in terms of practical computational performance. As illustrated in Figure 2, the three POPT schemes are briefly revisited through a simple case study, in which one of the four types of monomers (green ellipses marked with “A” or “D”) is appended to both ends of the system (represented by ellipses of other colors). To achieve this goal, the conventional SCF-based POPT must examine all (4×4) possible combinations in a single POPT cycle, selecting the optimal monomer based on (hyper)polarizabilities calculated using conventional FF for each combination. Since all SCF calculations at this stage are performed on the entire system (red and green ellipses), the computational cost of the conventional SCF-based POPT is significantly higher than that of the other two ELG-based POPT schemes. Among the remaining two ELG-based POPT schemes, the alternating POPT exhibits a greater efficiency advantage, which can be attributed to two main factors. First, in both alternating and simultaneous POPT schemes, the ELG-SCF calculations are restricted to the interactive space (orange and green ellipses). However, in the alternating POPT, a larger portion of the system lies far from the interactive space and can thus be treated using AO cutoff, leading to a significant reduction in computational cost. Second, since the alternating POPT proceeds in an alternating manner at the two terminals, the number of required combinations is reduced from (4×4) to (4+4), resulting in a further gain in efficiency.

**Figure 3:**
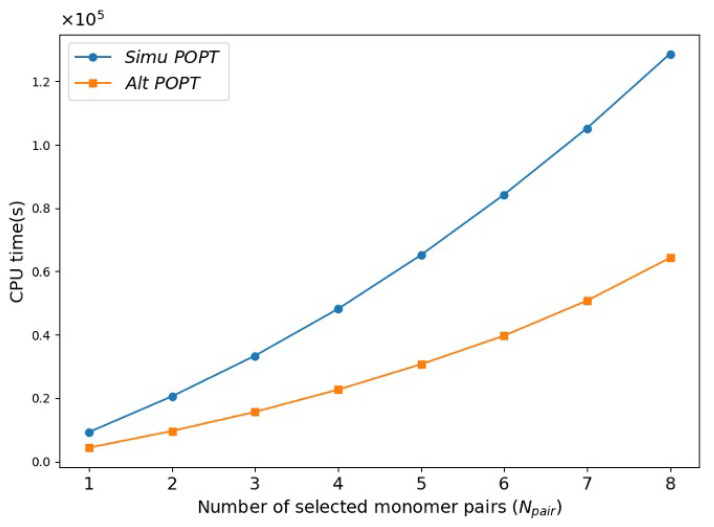
Comparison of the actual CPU time required by alternating POPT and simultaneous POPT for selecting $N_{pair}$ monomers in the minimization of $\alpha_{zz}$.

To further verify the efficiency of the alternating POPT in PFu systems, an $\alpha_{zz}$-minimizing simultaneous POPT was conducted for comparison. Notably, in the alternating POPT process proposed in this work, many combinations can be skipped compared to those considered in the simultaneous process [9]. The alternating POPT merely provides a way to design systems with good properties while controlling the computational cost, which is not achievable by the simultaneous POPT. It is assumed here that the simultaneous POPT yields the same PFu structure as that obtained from the $\alpha_{zz}$-minimizing alternating POPT to enable a comparison of computational efficiency. Figure 3 compares the cumulative actual CPU time required for the two $\alpha_{zz}$-minimizing POPT processes to select $N_{pair}$ pairs of furan monomers, highlighting a significant efficiency advantage of alternating POPT.

## 3. Materials and Methods

### 3.1. Alternating Elongation Method

**Figure 4:**
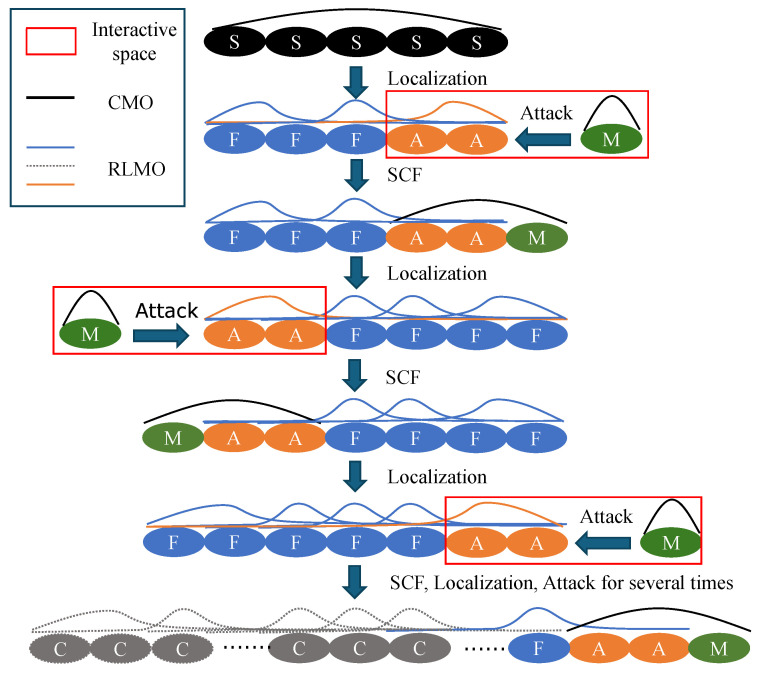
Diagram of the alternating elongation (ELG) process. Units in different regions are marked by ellipses in different colors. Canonical molecular orbitals (CMOs) and regional localized molecular orbitals (RLMOs) are represented by lines in distinct colors corresponding to each region.

Figure 4 schematically illustrates the alternating ELG approach in two directions [9]. The process begins with the starting cluster consisting of a few units, indicated by black ellipses labeled “S”. As an example, the elongation is first performed on the right terminal of the system. Its canonical molecular orbitals (CMOs) are obtained by SCF calculation and transformed into RLMOs [3] in two distinct regions: frozen and active. In the two regions, the units are represented by blue ellipses labeled “F” and orange ellipses labeled “A”, respectively. In Figure 4, the numbers of units in the frozen and active regions are set to 3 and 2, respectively, as an illustrative example. The active region is located at the right terminal for the next elongation. RLMOs in the frozen and active regions are represented by blue and orange lines, respectively, while the CMOs are depicted using black lines. Then, an attacking monomer, indicated by a green ellipse labeled “M”, is added to the terminal of the system where the active region is located, forming the interactive space indicated by the red box. After obtaining the wavefunction for the interactive space by ELG-SCF calculation, the starting cluster is elongated by one unit as the first ELG step.

In the subsequent ELG steps, analogous procedures are applied to the interactive space. The difference is that the terminal of the system to which the new attacking monomer is added is opposite to that in the previous ELG step. Several units in the frozen region near this terminal will be defined as the new active region. By alternately repeating the above ELG steps, the system can grow into the targeted structure.

During the ELG steps, the atomic orbital (AO) cutoff technique [39,40] is applied to units within the frozen region that are significantly distant from the active region. The transformation coefficients, from AOs associated with these units to active RLMOs (RLMOs in the active region), tend to approach zero. When constructing the RLMO–CMO-based Fock matrix for the ELG-SCF calculation of the interactive space, the contributions from these AOs are negligible. In this Fock matrix, RLMOs and CMOs are derived from the active region and the attacking monomer, respectively. The activation of the AO cutoff is controlled by a term $O=\sum_{\mu ,\nu \in F}\sum_{i\in S}\left|C_{\mu i}^{*}s_{\mu \nu }C_{\nu i}\right|$, which represents the coupling between the particular frozen region unit and the interactive space. *C* denotes the transformation matrix from AOs to RLMOs, and *s* is the AO overlap matrix. μ and ν represent the AOs of a certain frozen region unit F, and *i* denotes RLMOs belonging to the interactive space S. By default, when the value *O* is smaller than $10^{-5}$, the unit is subject to the AO cutoff procedure. The associated three- and four-center integrals can then be efficiently screened out and discarded [39,40]. The resulting ELG energy differs from the reference calculation without AO cutoff on the order of $5\times 10^{-7}$ a.u. This significantly reduces the computational cost of the ELG calculation while maintaining high accuracy in the total energy and the related (hyper)polarizability. Units affected by the AO cutoff procedure are shown in gray in Figure 4, and their corresponding RLMOs are represented by gray dashed lines.

### 3.2. Alternating POPT Process

**Figure 5:**
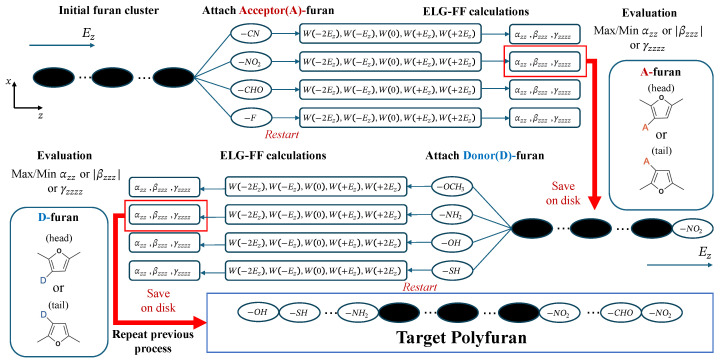
Diagram of the alternating property optimization (POPT) processes for designing polyfuran (PFu). The monomer selected for providing the optimal property in a specific POPT cycle is highlighted with a red box.

Figure 5 schematically illustrates the alternating POPT process [9] in this work. Before starting POPT, the electronic structures of an initial furan cluster under five different electric fields are calculated using the ELG method and stored in separate checkpoint files on disk. In the first POPT cycle, four acceptor-substituted furan (A-furan) monomers are sequentially added to the right terminal of the initial furan cluster. For each of the four generated PFus, ELG calculations under five different electric fields will be performed by restarting from the initial furan cluster’s checkpoint files.

(Hyper)polarizabilities for each case are calculated by the ELG-FF method [6,8], which treats a static homogeneous electric field *E* as an additional one-electron term in the Hamiltonian. All PFus are placed in the xz plane and aligned along the z-axis. Accordingly, the diagonal components of the (hyper)polarizability tensors, which are obtained through numerical differentiation as shown in Equations (2)–(4), can be used to represent the NLO responses.

(2) $$\begin{array}{cc} \alpha_{zz} & \approx \frac{1}{E_{z}^{2}}\left\{\frac{5}{2}W\left(0\right)-\frac{4}{3}\left[W\left(+E_{z}\right)+W\left(-E_{z}\right)\right]+\frac{1}{12}\left[W\left(+2E_{z}\right)+W\left(-2E_{z}\right)\right]\right\} \end{array}$$

(3) $$\begin{array}{cc} \beta_{zzz} & \approx \frac{1}{E_{z}^{3}}\left\{\left[W\left(+E_{z}\right)-W\left(-E_{z}\right)\right]-\frac{1}{2}\left[W\left(+2E_{z}\right)-W\left(-2E_{z}\right)\right]\right\} \end{array}$$

(4) $$\begin{array}{cc} \gamma_{zzzz} & \approx \frac{1}{E_{z}^{4}}\left\{-6W\left(0\right)+4\left[W\left(+E_{z}\right)+W\left(-E_{z}\right)\right]-\left[W\left(+2E_{z}\right)+W\left(-2E_{z}\right)\right]\right\} \end{array}$$

where W(E) is the ELG-calculated system’s total energy, in the presence of the electric field $E=-2E_{z},-E_{z},0,+E_{z},+2E_{z}$.

The monomer that meets specific criteria will be selected by evaluating the ELG-FF-calculated (hyper)polarizabilities. It is worth noting that monomers can be evaluated and selected according to a user-defined evaluation function, enabling a more flexible and diverse material design process. By modifying this function, such as defining it as the deviation between a calculated property (e.g., the band gaps) and a predefined target value, the design process can be directed toward materials with specific desired properties. We plan to develop and test this functionality further in future studies. Geometric coordinates and electronic structures of the new PFu with the selected monomer will be saved for the next POPT cycle. At the beginning of the next POPT cycle, four donor-substituted furan (D-furan) monomers will be added to the left terminal of the new PFu determined in the previous cycle, and the subsequent processing will be the same as before. By repeating the aforementioned cycles alternating between the left and right terminals, the design of a PFu with specific NLO properties can be accomplished.

It is noteworthy that all substituted PFus designed in this work are constructed in a head–tail connection pattern, with furan units of different orientations denoted as “head” or “tail”, as illustrated in Figure 5. When performing POPT at either terminal of the system, the orientation of the furan monomers involved in the current POPT cycle differs from that of the last unit already present at the corresponding terminal. Detailed information on the PFus and furan monomers will be provided in Appendix A.

## 4. Computational Details

A hybrid script combining Python and Bash was used to automate the alternating and simultaneous POPT procedures. Calculations for both ELG-FF and conventional FF methods were carried out at the HF/6-31G level, employing a modified GAMESS program [41]. An external electric field of 0.00025 atomic units was applied along the z-axis ($E_{z}$) during the computations. Given the novel nature of the ELG method, the HF/6-31G level was chosen to provide computational simplicity and facilitate method development, both of which are essential for verifying the reliability of the alternating POPT approach. In all ELG-FF and conventional FF calculations, the SCF process converges when the density change is less than $10^{-7}$ in absolute value. Both the alternating and simultaneous POPT processes described in Section 2.2 were computed on identical machines, each equipped with two Intel(R) Xeon(R) Gold 6226R CPUs, to compare CPU time.

The monomer geometric structure preparation process introduced in Appendix A is controlled by a Python script based on the quantum chemistry library PySCF [42,43]. Geometry optimization of non-substituted PFus was performed using Gaussian16 [44], and partial geometry optimizations were conducted using the PySCF library. All geometry optimizations were conducted at B3LYP [45]/6-311G(d,p) level.

## 5. Conclusions

Depending on the evaluation criteria, a total of six alternating POPT processes were conducted, with each POPT collectively adding 16 monomers. Formyl- and sulfhydryl-substituted furan monomers were preferentially selected in the $\alpha_{zz}$-maximizing POPT, whereas fluoro- and hydroxyl-substituted furan monomers were favored in the $\alpha_{zz}$-minimizing POPT. These observations may offer practical guidance for the future design of PFu-based NLO materials.

Compared to the POPT processes aimed at minimizing the PFus’ (hyper)polarizabilities ($\alpha_{zz}$, $|\beta_{zzz}|$, or $\gamma_{zzzz}$), the maximizing POPT processes exhibited significantly faster growth rates in their respective target properties, especially in the case of $|\beta_{zzz}|$. In the accuracy tests, the maximum relative error in (hyper)polarizability per atom during the alternating POPT process remained within 0.01% per atom, indicating a consistently low error level. In the efficiency tests, the alternating POPT continued to show a clear advantage in CPU time compared to the existing simultaneous POPT. Taken together, the alternating POPT approach maintained good performance when designing donor–acceptor-substituted PFu systems with specific NLO properties. This further supports its role as a reliable and efficient tool for designing systems with specific properties.

## Figures and Tables

**Table 1:** Donor–acceptor groups substituted in the furan monomers selected at each POPT cycle. The orientation of the furan monomer selected at each POPT cycle is indicated in parentheses.

POPT Cycle	Type	Max $\alpha_{\mathrm{zz}}$	Min $\alpha_{\mathrm{zz}}$	Max $|\beta_{\mathrm{zzz}}|$	Min $|\beta_{\mathrm{zzz}}|$	Max $\gamma_{\mathrm{zzzz}}$	Min $\gamma_{\mathrm{zzzz}}$
1 (head)	Acceptor	-CHO	-F	-NO_2_	-F	-CHO	-F
2 (head)	Donor	-SH	-OH	-NH_2_	-OCH_3_	-SH	-OH
3 (tail)	Acceptor	-CHO	-F	-CHO	-F	-CHO	-F
4 (tail)	Donor	-SH	-OH	-NH_2_	-SH	-SH	-NH_2_
5 (head)	Acceptor	-CHO	-F	-NO_2_	-F	-CN	-F
6 (head)	Donor	-SH	-OH	-NH_2_	-SH	-OCH_3_	-OH
7 (tail)	Acceptor	-CHO	-F	-CHO	-F	-CHO	-F
8 (tail)	Donor	-SH	-OH	-NH_2_	-SH	-OCH_3_	-OH
9 (head)	Acceptor	-CHO	-F	-NO_2_	-F	-CN	-F
10 (head)	Donor	-SH	-OH	-NH_2_	-SH	-OCH_3_	-OH
11 (tail)	Acceptor	-CHO	-F	-NO_2_	-F	-CHO	-F
12 (tail)	Donor	-SH	-OH	-NH_2_	-SH	-OCH_3_	-OH
13 (head)	Acceptor	-CHO	-F	-NO_2_	-F	-CN	-F
14 (head)	Donor	-SH	-OH	-NH_2_	-OH	-NH_2_	-OH
15 (tail)	Acceptor	-CHO	-F	-NO_2_	-F	-CHO	-F
16 (tail)	Donor	-SH	-OH	-NH_2_	-NH_2_	-OCH_3_	-OH

**Table 2:** Accuracy test result of alternating POPT. The error values are given in units of % per atom.

POPT Cycle	Err ($\alpha_{\mathrm{zz}}$)	Err ($\beta_{\mathrm{zzz}}$)	Err ($\gamma_{\mathrm{zzzz}}$)
1	3.83 $\times \;10^{-5}$	1.91 $\times \;10^{-4}$	1.30 $\times \;10^{-3}$
2	5.77 $\times \;10^{-5}$	1.35 $\times \;10^{-2}$	1.76 $\times \;10^{-3}$
3	8.20 $\times \;10^{-5}$	3.31 $\times \;10^{-3}$	2.10 $\times \;10^{-3}$
4	9.50 $\times \;10^{-5}$	1.23 $\times \;10^{-3}$	2.46 $\times \;10^{-3}$
5	1.03 $\times \;10^{-4}$	5.40 $\times \;10^{-4}$	2.70 $\times \;10^{-3}$
6	1.14 $\times \;10^{-4}$	1.79 $\times \;10^{-3}$	3.08 $\times \;10^{-3}$
7	1.30 $\times \;10^{-4}$	2.26 $\times \;10^{-3}$	3.56 $\times \;10^{-3}$
8	1.37 $\times \;10^{-4}$	7.93 $\times \;10^{-4}$	3.62 $\times \;10^{-3}$
9	1.37 $\times \;10^{-4}$	3.56 $\times \;10^{-4}$	3.59 $\times \;10^{-3}$
10	1.42 $\times \;10^{-4}$	1.08 $\times \;10^{-3}$	3.70 $\times \;10^{-3}$
11	1.52 $\times \;10^{-4}$	1.43 $\times \;10^{-3}$	3.91 $\times \;10^{-3}$
12	1.55 $\times \;10^{-4}$	3.23 $\times \;10^{-4}$	4.03 $\times \;10^{-3}$
13	1.51 $\times \;10^{-4}$	2.18 $\times \;10^{-5}$	3.83 $\times \;10^{-3}$
14	1.54 $\times \;10^{-4}$	1.01 $\times \;10^{-3}$	3.96 $\times \;10^{-3}$
15	1.58 $\times \;10^{-4}$	6.93 $\times \;10^{-4}$	3.99 $\times \;10^{-3}$
16	1.59 $\times \;10^{-4}$	1.08 $\times \;10^{-4}$	4.09 $\times \;10^{-3}$

**Figure A1:**
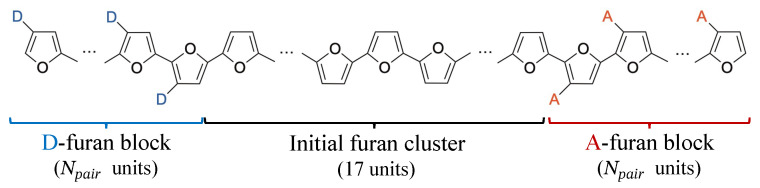
Structure of the target PFu.

**Figure A2:**
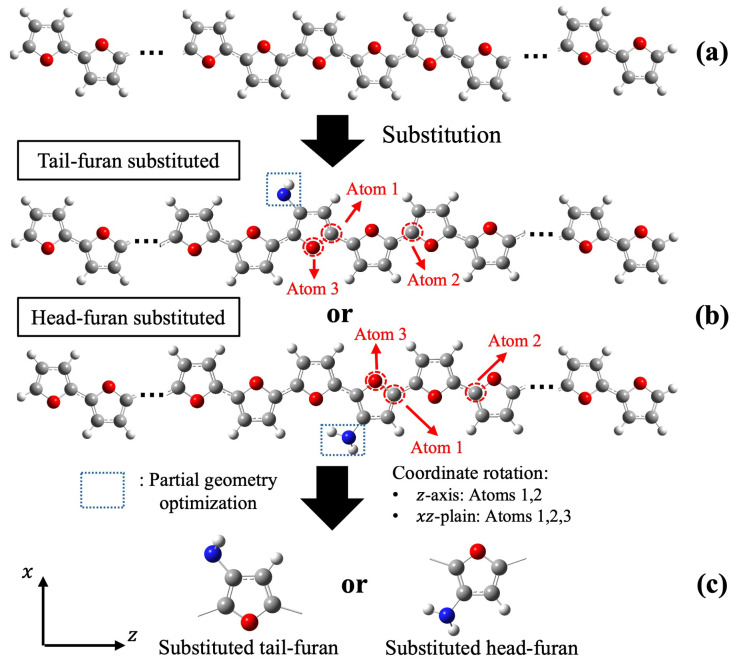
Preparation scheme of the substituted furan monomers. (**a**) Schematic illustration of the unsubstituted PFu structure; (**b**) Schematic illustration of the substituted PFu structure. The group that requires partial geometry optimization is indicated by a blue dashed box, and the atoms lying along the redefined z-axis and xz-plane after coordinate rotation are indicated by red dashed circles; (**c**) Schematic illustration of the target substituted furan monomer structure.

**Figure A3:**
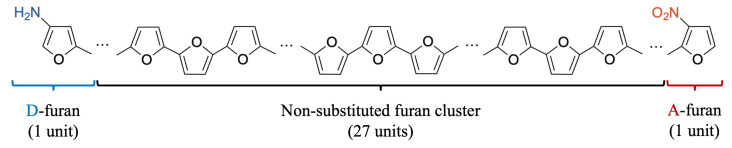
Structure of the sample PFu.

## Data Availability

The data presented in this work are available in the article.

## Abbreviations

The following abbreviations are used in this manuscript:

ELG	elongation
POPT	property optimization
FF	finite field
SCF	self-consistent field
NLO	non-linear optics
PFu	polyfuran
CMO	canonical molecular orbital
RLMO	regional localized molecular orbital
AO	atomic orbital
A-furan	acceptor-substituted furan
D-furan	donor-substituted furan

## Appendix A. Model Preparing

The target PFus of the alternating POPT process can be divided into three segments, all consisting of alternately connected head- and tail-furan units, as shown in Figure A1. The central segment is the initial furan cluster where the POPT process is initiated. On both sides of the initial cluster are blocks of A- and D-furan units. Each block contains $N_{pair}$ units, and the corresponding donor and acceptor groups on each substituted furan unit are indicated by blue “D” and red “A” letters, respectively.

The geometric structures of each D- or A-furan monomer are obtained through the process illustrated in Figure A2. A PFu consisting of a pair of head- and tail-furan units flanked by eight buffer units on each side is constructed. Its molecular structure is obtained through global geometry optimization, as shown in panel (a). Then, the central head- or tail-furan unit is substituted with a specific donor or acceptor group, and the positions of substitution are illustrated in panel (b). Taking the amino group as an example, the substitutions of the head- or tail-furan unit are exemplified by the two PFus displayed at the top and bottom, respectively. After partial geometry optimization for the substituted group, the coordinates of the substituted PFu will undergo a rotation using predefined atoms. As shown in panel (b), atoms 1 and 2 define the z-axis after rotation, while atoms 1, 2, and 3 together define the xz plane. Finally, the coordinates of the target head- or tail-furan unit containing the substituted group are recorded, as shown in panel (c).

In this work, methoxy -OCH_3_, amino -NH_2_, hydroxyl -OH, and sulfhydryl -SH groups are used as donors. Cyano -CN, fluoro -F, nitro -NO_2_, and formyl -CHO groups are used as acceptors. For all the groups mentioned above, the geometric structures of the corresponding substituted head- and tail-furan monomers are constructed, yielding a total of 16 configurations.

## Appendix B. Determination of the Interactive Space Size

In the ELG calculations presented in this work, the size of the interactive space (comprising the active region and one attacking monomer) was determined through testing. This ensures that the frozen RLMOs generated at each ELG step remain unaffected by the monomers added in subsequent ELG steps, while maintaining computational efficiency. The testing procedure has been described in detail in our previous work [9]. Here, we provide a brief summary for completeness.

A total of 13 ELG-FF calculations were carried out for the same sample PFu system shown in Figure A3, each using a different size of the interactive space. To provide a reference, a conventional FF calculation was also performed. The error per atom in the (hyper)polarizabilities was computed for each interactive space size using Equation (1) and is summarized in Table A1. When the number of units in the interactive space reaches 8, the error in the (hyper)polarizabilities drops below 0.01% per atom, which is already comparable to the accuracy achieved in our previous work [9]. While increasing the size of the interactive space beyond this point can further reduce the error, the benefit becomes marginal. For example, enlarging the size from 8 to 9 only reduces the error by $4\;\times \;10^{-3}\%$ per atom, while significantly increasing the computational cost, as all ELG calculations in the POPT procedure become more expensive. Therefore, in this work, we adopt an interactive space size of 8 units for all ELG calculations.

**Table A1:** Interactive space size test result. The error values are given in units of % per atom.

Number of Units	Err ($\alpha_{\mathrm{zz}}$)	Err ($\beta_{\mathrm{zzz}}$)	Err ($\gamma_{\mathrm{zzzz}}$)
2	5.74 $\times \;10^{-2}$	8.19 $\times \;10^{0}$	1.58 $\times \;10^{1}$
3	7.19 $\times \;10^{-2}$	1.82 $\times \;10^{1}$	1.93 $\times \;10^{1}$
4	1.16 $\times \;10^{-2}$	4.94 $\times \;10^{-1}$	5.64 $\times \;10^{-1}$
5	3.59 $\times \;10^{-3}$	8.00 $\times \;10^{-2}$	3.95 $\times \;10^{-2}$
6	1.11 $\times \;10^{-3}$	3.76 $\times \;10^{-2}$	2.15 $\times \;10^{-2}$
7	3.74 $\times \;10^{-4}$	1.69 $\times \;10^{-2}$	8.09 $\times \;10^{-3}$
8	1.38 $\times \;10^{-4}$	8.24 $\times \;10^{-3}$	3.11 $\times \;10^{-3}$
9	5.43 $\times \;10^{-5}$	3.46 $\times \;10^{-3}$	8.54 $\times \;10^{-4}$
10	2.18 $\times \;10^{-5}$	2.06 $\times \;10^{-3}$	4.11 $\times \;10^{-4}$
11	8.62 $\times \;10^{-6}$	1.04 $\times \;10^{-3}$	1.72 $\times \;10^{-4}$
12	3.44 $\times \;10^{-6}$	6.02 $\times \;10^{-4}$	3.27 $\times \;10^{-5}$
13	1.03 $\times \;10^{-6}$	3.47 $\times \;10^{-4}$	9.77 $\times \;10^{-5}$
14	3.66 $\times \;10^{-7}$	4.23 $\times \;10^{-5}$	2.06 $\times \;10^{-5}$
